# The First Identification of Homomorphic XY Sex Chromosomes by Integrating Cytogenetic and Transcriptomic Approaches in *Plestiodon elegans* (Scincidae)

**DOI:** 10.3390/genes15060664

**Published:** 2024-05-23

**Authors:** Wannan Xu, Taiyue Li, Jiahui Li, Siqi Liu, Xing Yu, Min Tang, Jingxiu Dong, Jianjun Liu, Xingjiang Bu, Xingquan Xia, Huaxing Zhou, Liuwang Nie

**Affiliations:** 1 The Anhui Provincial Key Laboratory of Biodiversity Conservation and Ecological Security in the Yangtze River Basin, College of Life Sciences, Anhui Normal University, Wuhu 241000, China; wnxu@ahnu.edu.cn (W.X.); 18297978035@163.com (T.L.); 13126892811@163.com (J.L.); lsq647666@outlook.com (S.L.); yuxing0511@163.com (X.Y.); mtang0216@outlook.com (M.T.); jxdong@cmpt.ac.cn (J.D.); buxingjiang@163.com (X.B.); quanwei@ahnu.edu.cn (X.X.); 2 Wuxi People’s Hospital Affiliated to Nanjing Medical University, Wuxi 214023, China; 3 Jiangsu Key Laboratory for Biodiversity and Biotechnology, College of Life Sciences, Nanjing Normal University, Nanjing 210098, China; ahnubioliu@outlook.com; 4 Anhui Key Laboratory of Aquaculture & Stock Enhancement, Fisheries Research Institution, Anhui Academy of Agricultural Sciences, Hefei 230041, China

**Keywords:** *Plestiodon elegans*, karyotype, RNAseq, sex markers, sex chromosomes

## Abstract

The sex chromosomes of skinks are usually poorly differentiated and hardly distinguished by cytogenetic methods. Therefore, identifying sex chromosomes in species lacking easily recognizable heteromorphic sex chromosomes is necessary to fully understand sex chromosome diversity. In this paper, we employed cytogenetics, sex quantification of genes, and transcriptomic approaches to characterize the sex chromosomes in *Plestiodon elegans*. Cytogenetic examination of metaphases revealed a diploid number of 2n = 26, consisting of 12 macrochromosomes and 14 microchromosomes, with no significant heteromorphic chromosome pairs, speculating that the sex chromosomes may be homomorphic or poorly differentiated. The results of the sex quantification of genes showed that Calumenin (*calu*), COPI coat complex subunit γ 2 (*copg2*), and Smoothened (*smo*) were at half the dose in males as in females, suggesting that they are on the X chromosome. Transcriptomic data analysis from the gonads yielded the excess expression male-specific genes (n = 16), in which five PCR molecular markers were developed. Restricting the observed heterozygosity to males suggests the presence of homomorphic sex chromosomes in *P. elegans*, XX/XY. This is the first breakthrough in the study of the sex chromosomes of *Plestiodon*.

## 1. Introduction

Reptiles have different strategies for sex determination, ranging from temperature-dependent sex determination (TSD) to genetic sex determination (GSD) [1,2]. The sex chromosomes of GSD species can be classified as heteromorphic and homomorphic [3]. Cytogenetics is a common method for identifying heteromorphic sex chromosomes, such as Giemsa staining, Ag-NOR banding, C-banding, G-banding, and FISH technology, whereas homomorphic sex chromosomes need to be identified by molecular biology methods, such as sex quantification of genes (qPCR), to identify X/Z-linked genes; RNAseq, amplified fragment length polymorphism (AFLP), restriction site-associated DNA sequencing (RADseq), and whole genome subtraction (ISWGS) are used to identify Y/W-linked genes [2]. Cytogenetics combined with molecular biology is a prevalent strategy for sex chromosome identification [4,5].

There are about 1760 species of skinks, accounting for 15% of the reptile species diversity [6]. They vary in chromosome number from 2n = 22 to 2n = 36, include both macrochromosomes and microchromosomes [5,7,8], and show genus conservation [8,9,10]. Many careful cytogenetic studies on skinks were conducted in the past, but sex chromosomes were rarely identified [8], and only 39 species have been reported with sex chromosomes to date [3,5,8,11]. The majority of skinks are XX/XY, and the only report of ZZ/ZW sex chromosomes in skinks was based on size difference between the putative Z and W in *Scincella melanosticta* (Boulenger, 1887) [12]. The distribution of sex chromosomes is most complex in *Scincella* Mittleman, 1950, which is XX/XY in *S. assata* (Cope, 1864) and *S. cherriei* (Cope, 1864) and ZZ/ZW in *S. melanosticta* (Boulenger, 1887), while there are population differences in the sex chromosomes of *S. lateral* (Say, 1822) (XX/XY for the southeastern U.S. population and X1X1X2X2/X1X2Y for a unique population from Texas) [8]. *Plestiodon* Duméril & Bibron, 1839 is an important component of skinks, with 51 species. Except for *P. anthracinus* (Baird, 1849) (2n = 24, 12 macrochromosomes and 12 microchromosomes), the remaining 16 species of *Plestiodon* with published karyotypes are 2n = 26 (12 macrochromosomes and 14 microchromosomes) [7,13]; regrettably, the group has no cases of sex chromosomes so far.

*P. elegans* (Boulenger, 1887) is widely distributed in southern China [14,15], and it has a chromosome number of 2n = 26 [16], but the sex chromosomes are still unknown. In this paper, we employ cytogenetics, sex quantification of genes, and transcriptomic data to characterize the sex chromosomes. This study paves the way for the molecular biology of sex identification in *P. elegans* and adds data to the database of the dim sex chromosomes of skinks.

## 2. Materials and Methods

### 2.1. Sample Collection

All *P. elegans* used in this study were collected from a farm in Yihuang (116°13′ N, 27°28′ E), Fuzhou, China. They were transferred to the laboratory and kept under standard conditions for one week before the experimentation.

### 2.2. Chromosome Preparation and Staining

Chromosomes were prepared using bone marrow and testes tissue, respectively, following the method of Patawang et al. [7] and Wang et al. [17]. The slides were routinely stained for 30 min using 10% Giemsa’s solution [18]. Ag-NOR banding was performed according to Howell and Black [19]. Chromosome images were acquired using a DM1000 microscope (Leica, Wetzlar, Germany). We measured chromosome length and estimated the relative length (RL) and centromeric index (CI) (Supplementary Table S1). All data were used in karyotyping and diagramming [20].

### 2.3. Sex Quantification of Genes

In GSD species, genes on the nonrecombining region on the Z chromosome in males (ZZ) and the X chromosome in females (XX) have twice as many copies as those in females (ZW) and males (XY). In contrast, genes on the autosomes have equal copies in males and females. So, the differences in the copy number of genes between the sexes can be determined by qPCR, which allows for the identification of Z- (or X-) linked genes [21,22,23]. This method has been successfully applied to lizards of Lacertidae [24], Agamidae [25], Dactyloidae [26], Scincidae [3], etc. *Scincus scincus* (Linnaeus, 1758) (with heterozygous XX/XY chromosomes [27]) and *P. elegans* belong to the subfamily Scincinae, and we hypothesize that these species have homologous X chromosomes. Based on the X-linked and autosomal genes of *S. scincus* reported by Kostmann et al. [3], we performed a gene dosage analysis of *P. elegans*. Genes and primers can be found as reported in Kostmann et al. [3], or see Table S2. The qPCR was carried out in a LightCycler 96 (Roche, Mannheim, Germany) with all samples run in triplicate. The gene dosage of each studied gene was calculated from crossing point values (Cps) and was subsequently normalized to the gene dosage of the single copy gene *ef1a1* from the same DNA sample, based on the following equation: R = 2*_ef__1a__1_*^Cp *ef*^*^1a1^*/2_gene_^Cp gene^. Finally, the relative gene dosage ratio (r) between the two sexes for each gene and each species was obtained by dividing the average gene dosage in the female by the average dosage in the male, as r = R_male_/R_female_. The relative gene dosage ratio (r) is close to 0.5 for X-linked genes, 1.0 for (pseudo-)autosomal genes, and 2.0 for Z-linked genes [21].

### 2.4. RNA Sequencing and Identification of Sex Markers

The development of sex markers using transcriptomic data was based on the method of Saunders et al. [28] with slight modifications. Gonads from 3 males and 3 females were taken, total RNA was extracted using TRIzol^®^, and the constructed sequencing library was sequenced (150 bp × 2) using the Illumina NovaSeq 6000 platform (Shanghai BIOZERON Co., Ltd., Shanghai, China). Reads were trimmed using Trimmomatic (v0.3.2, Sliding window: 4:15, Minlen: 75). De novo assembly was performed using Trinity (v2.8.4) default parameters, and clean reads from each sample were mapped to the assembled sequences. The assembled sequences were aligned with the NR, Swiss-Prot, eggNOG, KEGG, and GO databases to obtain annotated information about the predicted genes. The reads of each library were mapped to the assemblies using Bowtie (v2.3.4.3) and the abundance of transcripts was estimated using RSEM (v1.3.1). Gene expression was calculated using reads per kilobase of exon per million mapped reads (RPKM). Here, we define RPKM ≥ 1 and =0 as expressed and unexpressed, respectively, to screen for sex-specific genes [29].

We screened sixteen male-specific genes and one female-specific gene (putative Y-linked genes and a W-linked gene) based on the RPKM, and then designed 28 primer pairs (Table S3) for validation of sex markers using Primer (v3.2.3.1). These primers were individually evaluated with PCR against the genomes of *P. elegans* of known sex, and the PCR products were separated on agarose gels. The sex specificity of the amplified products was identified based on the presence or absence of distinct bands. In addition, control primers (18S rDNA, Table S3) referencing the 18S ribosomal RNA gene (GenBank: AY859624.1) of *Anolis carolinensis* (Voigt, 1832, Anolidae) were designed. PCR conditions were as follows: denaturation at 95 °C for 5 min, followed by 35 cycles (denaturation at 95 °C for 30 s, annealing at 59 °C for 30 s, extension at 72 °C for 40 s), and extension at 72 °C for 10 min. The PCR products were subjected to Sanger sequencing to confirm their consistency with the sequences of the related transcripts.

In addition, we tested the suitability of these sex markers in *P. capito* (Bocourt, 1879) and *P. chinensis* (Gray, 1839) using PCR.

## 3. Results

### 3.1. Karyotypes of P. elegans

**Figure 1:**
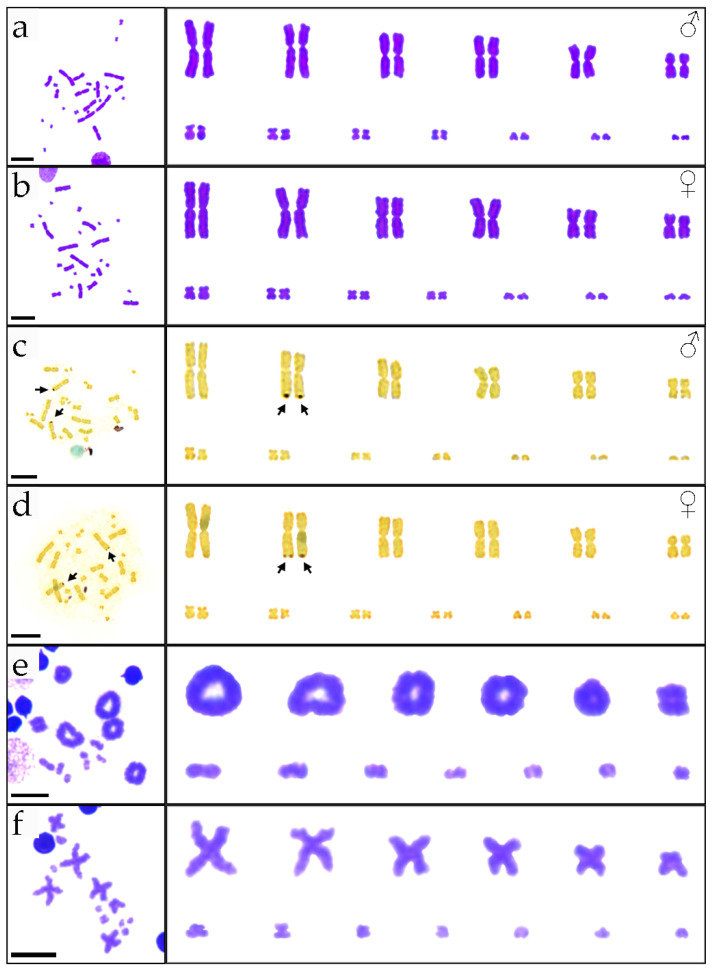
Karyotypes of *P. elegans*. Mitotic metaphase chromosome spreads and karyotypes by Giemsa staining (**a**,**b**) and Ag-NOR banding (**c**,**d**). Meiotic metaphase chromosome spreads and karyotypes at metaphase I (**e**) and metaphase II (**f**) by Giemsa staining. Male: (**a**,**c**,**e**,**f**); female: (**b**,**d**). Arrows indicate NORs. The scale bar indicates 10 µm.

The diploid chromosome number of *P. elegans* is 2n = 26, consisting of 12 macrochromosomes and 14 microchromosomes, and the macrochromosomes were demarcated from the microchromosomes in a bimodal karyotype (Figure 1a,b). The chromosome composition was 10 large metacentric, 2 medium metacentric, 8 small metacentric, and 6 small telocentric chromosomes, with NF = 46, which is consistent with previous reports [16]. Dark-colored bands (NOR positions) are shown in the telomere region on the long arm of chromosome pair 2 (Figure 1c,d). We found that during metaphase I on meiosis, the homologous chromosomes showed synapsis, which can be defined as the 13 bivalents and 13 haploid chromosomes in metaphase II as diploid species (Figure 1e,f).

We measured the lengths of chromosomes during the metaphases. The largest chromosomes were 17 times larger than the smallest chromosomes, and large chromosomes accounted for 76% of the total karyotype chromosome length (Table S1). The karyotype formula for *P. elegans* can be derived as 2n = 26 = L^m^_10_ + M^m^_2_ + S^m^_8_ + S^t^_6_. Cytogenetic results did not show sex chromosomes that were heteromorphic in size or structure, and we hypothesize that sex chromosomes in *P. elegans* are either homomorphic or poorly differentiated.

### 3.2. The X-Linked Genes of P. elegans

**Figure 2:**
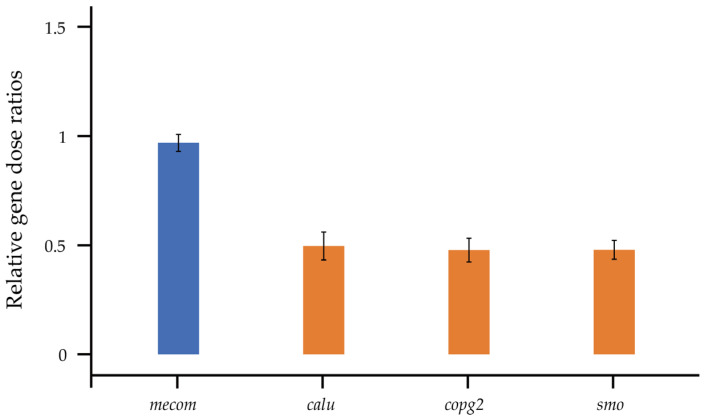
Relative gene dose ratios between males and females for genes in *P. elegans*. The value 1.0 is expected for (pseudo-)autosomal genes (blue bar), whereas the value 0.5 is consistent with X-specificity (orange bars).

In the 13 genes used for qPCR, only 5 genes (*calu*, *copg2*, *smo*, *ef1a1*, and *mecom*, Table S2) showed single peaks in the melting curves, suggesting that these 5 genes were successfully amplified in *P. elegans*. Using *ef1a1* as the reference gene, the gene dose ratios of the other four genes were calculated based on the Cp values, and the average value was used as the final result to predict the sex chromosome type of *P. elegans* (Table S4). qPCR results (Figure 2) showed that the relative gene dose ratio of *mecom* was 1, indicating that the gene might be located in the (pseudo-)autosomes. *Calu*, *copg2*, and *smo* had quantitative values in males that were half those of females, indicating that they are on the X chromosome and not on the Y chromosome. It is noteworthy that the same pattern of these three X-linked genes is present in 13 other Scincidae species [3]. The sequence information of *mecom*, *calu*, *copg2*, and *smo* of *P. elegans* is shown in Table S5.

### 3.3. Transcriptome Analysis of Gonads and Development of Sex Markers in P. elegans

**Figure 3:**
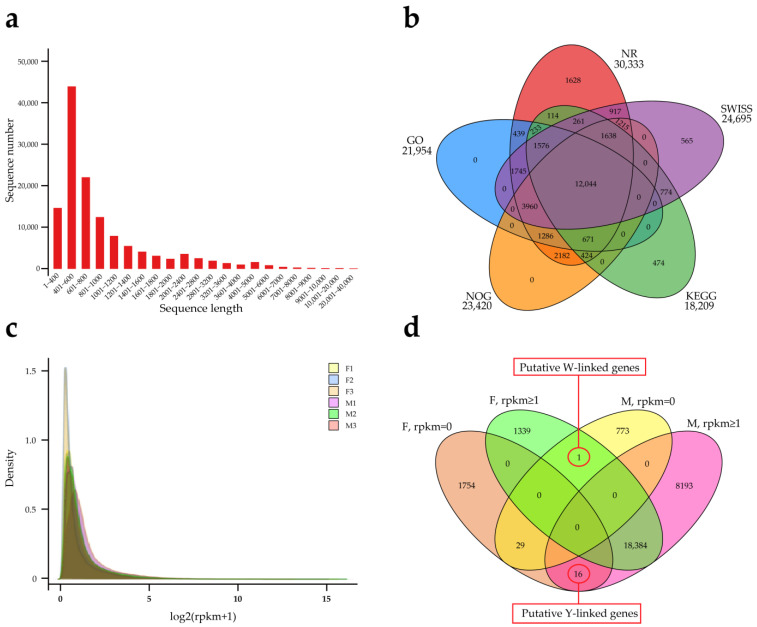
Transcriptomes in the gonads and sex markers of *P. elegans*. Assembly sequence length distribution (**a**), gene function annotation statistics (**b**), expression level density curves for different samples (**c**), and RPKM ≥ 1 and =0 statistics for genes in different sexes (**d**).

RNAseq built and sequenced six libraries (3♂, 3♀), generating 488,010,098 raw sequences (Table S6), which were filtered to yield 484,760,322 clean reads (Table S7). The assembly from scratch produced 164,575 isoforms, which were clustered into 126,511 unigenes (Table S8). The length of the assembled sequences is shown in Figure 3a, and the longest unigene is 30,162 bp. The clean reads of each sample were mapped with the assembly results, and the ratios were more than 91% (Table S9), which indicated that the assembly results were credible. A total of 32,142 unigenes were annotated by five databases (Figure 3b), accounting for 25% of the total unigenes. Figure 3c demonstrates the expression probability density distribution, and the related genes have gender expression differences. Clustering the genes in the different samples according to RPKM (Table S10), 16 genes were expressed only in males, which were defined as putative Y-linked genes (Figure 3d and Table S11). The excess of male-specific genes provides further evidence for the existence of the Y chromosome [11].

**Figure 4:**
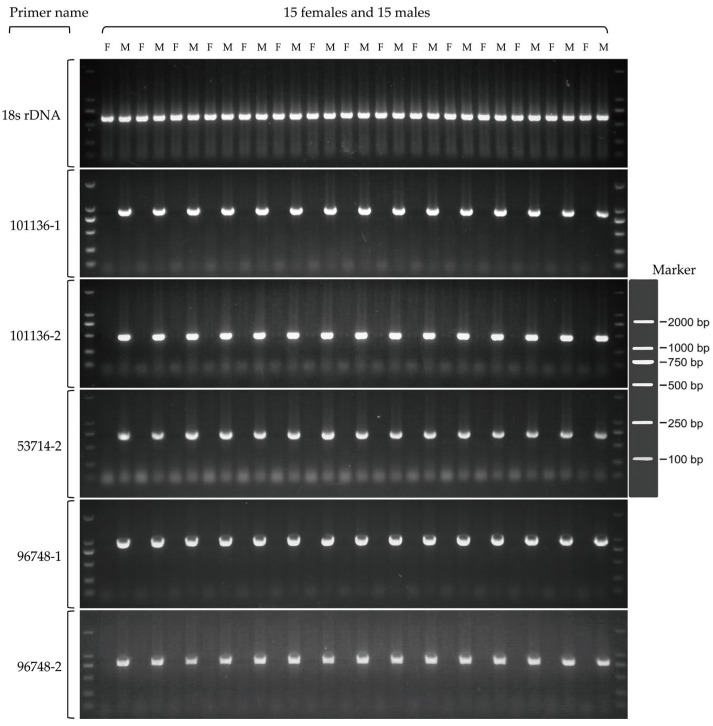
Validation of 5 male markers in *P. elegans* using a panel of 15 male and 15 female individuals of confirmed phenotypic sex. 18s rDNA as control.

Chromosomes amplified in a sex-specific manner, and they produced a single distinct band only in male samples. The PCR products of these five primer pairs can be used as molecular markers for sex identification, and the genes associated with them are Y-linked genes (Figure 4, Table S5). We tested these sex markers on *P. capito* and *P. chinensis*, and none of the bands amplified (Figure S1). Sanger sequencing of these markers confirmed the amplification of the target fragments (Figure S2). Notably, among these five male markers, 101136-1 and 101136-2 showed excellent similarity to the reptilian Ubiquitin-conjugating enzyme E2 H (*UBE2H*) sequence. In addition, the Y chromosomes of *Bassiana duperreyi* (Hutchinson et al., 1990, Scincidae) [30] and *Eulamprus heatwolei* (Wells & Wellington, 1983, Scincidae) [31] also contain partial sequences of *UBE2H*, suggesting that the Y chromosomes of these three species of skinks have homologous regions and may have homologous sex chromosomes.

## 4. Discussion

In conclusion, using cytogenetic and molecular biological methods, we demonstrated the presence of homomorphic XX/XY sex chromosomes in *P. elegans*. This is the first species in *Plestiodon* to have its sex chromosomes successfully identified and adds data to the small database of sex chromosomes in skinks.

One of the reasons for the sparse database on the sex chromosomes of skinks is that the majority of members of the group possess homomorphic chromosomes, which are difficult to detect even by careful cytogenetic studies [8]. Two main molecular techniques, qPCR and RADseq, have the power to provide a qualitative/quantitative identification of sex-specific sequences, also uncovering the occurrence of GSD and sex chromosome systems in species with homomorphic sex chromosomes [2]. Rovatsos and Kratochvíl [21] highlighted the fact that qPCR can identify the sexes of about 4000 species (nearly 50% of the recent species of reptiles), with RADseq being applied in a relatively limited number of cases [5]. However, in many non-model species, the identification of sex-specific sequences and the development of PCR-based sex identification methods is challenging without additional genomic resources [5]. In this study, we developed five Y chromosome markers using transcriptome, and RNAseq-PCR is a user-friendly and inexpensive method [28]. Gonads were used as experimental materials, and Y-linked genes were identified based only on the principle that genes have sex-specific expression differences. The method does not require a reference genome and is suitable for biological studies where a reference genome is unavailable or incomplete [28,32]. It is important to note that there were a large number of false positives in the results of the analysis of the unreferenced transcriptome [33], which may have contributed to the success rate of only 18% in this study. However, we finally identified the Y chromosome markers using PCR. Therefore, PCR validation was effective in eliminating false positives caused by sequence incompleteness of the unreferenced transcriptome and sex differences in gene splicing [34]. Similarly, Lamatsch et al. [32] and Saunders et al. [28] identified Y chromosome markers using RNAseq-PCR for *Gambusia affinis* (Baird & Girard, 1853, Poeciliidae) and *Carinascincus ocellatus* (Gray, 1845, Scincidae), respectively. The most conservative conclusions suggest that the development of sex markers for PCR assays using only transcriptomic data presupposes that the gonads served as the material, and that nonrecombinant sequences of the Y/W chromosomes were expressively active. 

Two recent studies shed light on a shared conserved XX/XY sex chromosome system in most skinks [2,3,11]. In the first study, the authors used genome coverage analysis to identify the X-linked genes of *S. Scincus* and qPCR-tested these X-linked genes among 13 species of the family Scincidae, demonstrating that skinks have shared the same homologous XX/XY sex chromosomes across their wide phylogenetic spectrum for at least 85 million years [3]. The second study used RADseq to identify Y-linked genes in *Liopholis whitii* (Lacépède, 1804) and detected sex differences in the amplification of these genes in 13 species of the Egernia group, demonstrating that the XX/XY sex-determination system is conserved in this group [11]. The results of the present study show that some of the X-linked genes from the first study mentioned above also apply to *P. elegans*. In addition, we demonstrated the presence of *UBE2H* on the Y chromosome of *P. elegans* (this study), *B. duperreyi*, and *E. heatwolei*, which indicates that they have homologous sex chromosomes. Both *P. capito* and *P. chinensis* belong to the genus *Plestiodon* [6], but their sex chromosomes have not been reported. Zheng and Wiens [35] suggested that *P. chinensis* diverged by ~48 Mya (CI: 29.2–60.3), and that *P. capito* and *P. elegans* separated before ~21.8 Mya (CI: 0–35.9). The five Y chromosome markers that we screened for in *P. elegans* do not apply to *P. capito* and *P. chinensis*, and the sex chromosomes of these three species may have mutated during evolution [3,36,37], thus limiting the versatility of the sex markers. Although the karyotypes of these three skinks are highly conserved (2n = 26, 12 macrochromosomes and 14 microchromosomes) [38], there are differences in the location of the NORs, which are located on chromosome pair 2 (macrochromosomes) in *P. elegans* and *P. chinensis* [39], and on chromosome pair 7 (microchromosomes) in *P. capito* [38]. It should be emphasized that none of their NORs are sex-differentiated.

The identification of sex markers is of great practical value in many situations, including ecological studies [40], the conservation of threatened or endangered species [41], captive breeding [42], and the identification of the sex of embryos [43]. We have demonstrated that *calu*, *copg2*, and *smo* are located in the X chromosome heterologous region of *P. elegans*; there are reports demonstrating that they play a key role in gonadal development in several species (i.e., bovine, human, and white shrimp) [3,44,45]. In addition, we demonstrated the presence of *UBE2H* on the Y chromosome of *P. elegans*. Ubiquitin-conjugating enzymes are encoded by a family of highly conserved genes involved in post-translational processes targeting abnormal or short-lived proteins for degradation [46]. Studies have shown that members of the Ubiquitin-conjugating enzymes family are involved in testicular developmental processes, such as testis-specific UBC4-testis in rats [47] and UBE2R-testis in *Ciona intestinalis* (Linnaeus, 1767, Cionidae) [48]. The present study has only demonstrated that these genes are present on the sex chromosomes of *P. elegans*, and whether or not they play an important role in sex determination still requires further studies.

Future research on *P. elegans* will focus on two areas: chromosomal localization using developed molecular sex markers as probes and deciphering the function of identified sex chromosome-linked genes in regulating gonadal development.

## Data Availability

The data presented in this study are available in the article and Supplementary Material. RNAseq data can be obtained from the Transcriptome Shotgun Assembly project DDBJ under accession number PRJNA1098909.

## Supplementary Materials

The following supporting information can be downloaded at: https://www.mdpi.com/article/10.3390/genes15060664/s1, Figure S1: The Y chromosome markers of *P. elegans* cannot be amplified in *P. capito* and *P. chinensis*. 18s rDNA as control; Figure S2: Sequence alignment results of Y chromosome markers with Y-linked genes; Table S1: The mean length of short arm chromosome (Ls), length of long arm chromosomes (Ll), total length of chromosomes (LT), relative length (RL), centromeric index (CI), and standard deviation (SD) of 20 metaphases of male and female *P. elegans*, 2n = 26; Table S2: Genes and primers involved in qPCR; Table S3: Primer pairs for the amplification of male-specific sequences for *P. elegans*. Primer pair 18s rDNA was the control; Table S4: Dosage ratios of related genes in the 3 sex groups; Table S5: Sequence information for genes and markers; Table S6: Raw data statistics; Table S7: Post-quality control statistics; Table S8: Statistics of splicing results; Table S9: Mapping ratio statistics; Table S10: RPKM statistics; Table S11: Putative sex-specific genes.

## References

[1] Martínez-Juárez A., Moreno-Mendoza N. (2019). Mechanisms related to sexual determination by temperature in reptiles. J. Therm. Biol..

[2] Thépot D. (2021). Sex chromosomes and master sex-determining genes in turtles and other reptiles. Genes.

[3] Kostmann A., Kratochvíl L., Rovatsos M. (2021). Poorly differentiated XX/XY sex chromosomes are widely shared across skink radiation. Proc. R. Soc. B.

[4] Singchat W., Sillapaprayoon S., Muangmai N., Baicharoen S., Indananda C., Duengkae P., Peyachoknagul S., O’Connor R.E., Griffin D.K., Srikulnath K. (2020). Do sex chromosomes of snakes, monitor lizards, and iguanian lizards result from multiple fission of an “ancestral amniote super-sex chromosome”?. Chromosome Res..

[5] Mezzasalma M., Guarino F.M., Odierna G. (2021). Lizards as model organisms of sex chromosome evolution: What we really know from a systematic distribution of available data?. Genes.

[6] Uetz P. (2016). The reptile database turns 20. Herpetol. Rev..

[7] Patawang I., Tanomtong A., Jumrusthanasan S., Khongcharoensuk H., Kaewsri S., Pinthong K. (2017). Cytogenetic of skink (Reptilia, Scincidae) from Thailand: II: Chromosome analyses of stripe tree skink (*Lipinia vittigera*). Cytologia.

[8] Kostmann A. (2021). Evolution of Sex Determination in Skinks and Related Lineages. Ph.D. Thesis.

[9] Gordon D.H., Haacke W.D., Jacobsen N.H.G. (1987). Chromosomal studies of relationships in Gekkonidae, Chamaeleonidae and Scincidae in South Africa. J. Herpetol. Assoc. Afr..

[10] Caputo V., Odierna G., Aprea G. (1993). Karyological comparison of *Sphenops sepsoides*, *Chalcides chalcides*, and *C. ocellatus* (Reptilia: Scincidae): Taxonomic implications. Copeia.

[11] Bouffet-Halle A., Yang W., Gardner M.G., Whiting M.J., Wapstra E., Uller T., While G.M. (2022). Characterisation and cross-amplification of sex-specific genetic markers in Australasian Egerniinae lizards and their implications for understanding the evolution of sex determination and social complexity. Aust. J. Zool..

[12] Patawang I., Chuaynkern Y., Supanuam P., Maneechot N., Pinthong K., Tanomtong A. (2018). Cytogenetics of the skinks (Reptilia, Scincidae) from Thailand; IV: Newly investigated karyotypic features of *Lygosoma quadrupes* and *Scincella melanosticta*. Caryologia.

[13] Hardy L.M., Raymond L.R., Harris S. (2017). The karyotype of *Plestiodon anthracinus* (Baird, 1850) (Sauria: Scincidae): A step toward solving an enigma. Southeast. Nat..

[14] Du W.G., Yan S.J., Ji X. (2000). Selected body temperature, thermal tolerance and thermal dependence of food assimilation and locomotor performance in adult blue-tailed skinks, *Eumeces elegans*. J. Therm. Biol..

[15] Du W.G., Lu Y.W., Shu L., Bao Y.X. (2007). Thermal dependence of food assimilation and locomotor performance in juvenile blue-tailed skinks, *Eumeces elegans*. Anim. Biol..

[16] Yongzhang L., Yongpu Z. (2003). Comparison of karyotypes of different geographical populations of *Eumeces elegans*. Chin. J. Zool..

[17] Wang C., Tang X., Xin Y., Yue F., Yan X., Liu B., An B., Wang X., Chen Q. (2015). Identification of sex chromosomes by means of comparative genomic hybridization in a lizard. Eremias multiocellata. Zool. Sci..

[18] Chooseangjaew S., Tanyaros S., Maneechot N., Buasriyot P., Getlekha N., Tanomtong A. (2017). Chromosomal characteristics of the tropical oyster, *Crassostrea belcheri* Sowerby, 1871 (Ostreoida, Ostreidae) by conventional and Ag-NOR banding techniques. Cytologia.

[19] Howell W.M., Black D.A. (1980). Controlled silver-staining of nucleolus organizer regions with a protective colloidal developer: A 1-step method. Experientia.

[20] Deshmukh P.V., Yadav S.R., Lekhak M.M. (2023). Karyomorphological study in *Ledebouria botryoides* (Asparagaceae). Cytologia.

[21] Rovatsos M., Kratochvíl L. (2017). Molecular sexing applicable in 4000 species of lizards and snakes? From dream to real possibility. Methods Ecol. Evol..

[22] Rovatsos M., Farkačová K., Altmanová M., Johnson Pokorná M.J., Kratochvíl L. (2019). The rise and fall of differentiated sex chromosomes in geckos. Mol. Ecol..

[23] Pensabene E., Kratochvíl L., Rovatsos M. (2020). Independent evolution of sex chromosomes in eublepharid geckos, a lineage with environmental and genotypic sex determination. Life.

[24] Rovatsos M., Vukić J., Altmanová M., Pokorná M.J., Moravec J., Kratochvíl L. (2016). Conservation of sex chromosomes in lacertid lizards. Mol. Ecol..

[25] Rovatsos M., Pokorná M., Altmanová M., Kratochvíl L. (2014). Cretaceous park of sex determination: Sex chromosomes are conserved across iguanas. Biol. Lett..

[26] Rovatsos M., Altmanová M., Pokorná M., Kratochvíl L. (2014). Conserved sex chromosomes across adaptively radiated Anolis lizards. Evolution.

[27] Caputo V., Odierna G., Aprea G. (1994). A chromosomal study of *Eumeces* and *Scincus*, primitive members of the Scincidae (Reptilia, squamata). Ital. J. Zool..

[28] Saunders P.A., Ferre-Ortega C., Hill P., Simakov O., Ezaz T., Burridge C.P., Wapstra E. (2023). Using a handful of transcriptomes to detect sex-linked markers in a lizard with homomorphic sex chromosomes. BioRxiv.

[29] Zeng C., Fukunaga T., Hamada M. (2018). Identification and analysis of ribosome-associated lncRNAs using ribosome profiling data. BMC Genom..

[30] Dissanayake D.S.B., Holleley C.E., Hill L.K., O’Meally D., Deakin J.E., Georges A. (2020). Identification of Y chromosome markers in the eastern three-lined skink (*Bassiana duperreyi*) using in silico whole genome subtraction. BMC Genom..

[31] Cornejo-Páramo P., Dissanayake D.S.B., Lira-Noriega A., Martínez-Pacheco M.L., Acosta A., Ramírez-Suástegui C., Méndez-De-La-Cruz F.R., Székely T., Urrutia A.O., Georges A. (2020). Viviparous reptile regarded to have temperature-dependent sex determination has old XY chromosomes. Genome Biol. Evol..

[32] Lamatsch D.K., Adolfsson S., Senior A.M., Christiansen G., Pichler M., Ozaki Y., Smeds L., Schartl M., Nakagawa S. (2015). A transcriptome derived female-specific marker from the invasive western mosquitofish (*Gambusia affinis*). PLoS ONE.

[33] Small C.M., Carney G.E., Mo Q., Vannucci M., Jones A.G. (2009). A microarray analysis of sex-and gonad-biased gene expression in the zebrafish: Evidence for masculinization of the transcriptome. BMC Genom..

[34] Trabzuni D., Ramasamy A., Imran S., Walker R., Smith C., Weale M.E., Hardy J., Ryten M., North American Brain Expression Consortium (2013). Widespread sex differences in gene expression and splicing in the adult human brain. Nat. Commun..

[35] Zheng Y., Wiens J.J. (2016). Combining phylogenomic and supermatrix approaches, and a time-calibrated phylogeny for squamate reptiles (lizards and snakes) based on 52 genes and 4162 species. Mol. Phylogenetics Evol..

[36] Ezaz T., Sarre S.D., O’Meally D., Marshall Graves J.A., Georges A. (2010). Sex chromosome evolution in lizards: Independent origins and rapid transitions. Cytogenet. Genome Res..

[37] Alam S.M.I., Sarre S.D., Gleeson D., Georges A., Ezaz T. (2018). Did lizards follow unique pathways in sex chromosome evolution?. Genes.

[38] Xu W.N., Zhu W.R. (2024). First karyological analysis of *Plestiodon capito* (Squamata: Scincidae). Cytologia.

[39] Guo C., Dong Y.W. (1988). A comparative study on the karyotypes and Ag-stained NORs of two species of wild skinks from Huang Shan. Hereditas.

[40] Ferguson-Smith M. (2006). The evolution of sex chromosomes and sex determination in vertebrates and the key role of DMRT1. Sex. Dev..

[41] Literman R., Radhakrishnan S., Tamplin J., Burke R., Dresser C., Valenzuela N. (2017). Development of sexing primers in *Glyptemys insculpta* and *Apalone spinifera* turtles uncovers an XX/XY sex-determining system in the critically-endangered bog turtle *Glyptemys muhlenbergii*. Conserv. Genet. Resour..

[42] Sulandart S.R.I., Zein M.S.A. (2012). Application of two molecular sexing methods for Indonesian bird species: Implication for captive breeding programs in Indonesia. HAYATI J. Biosci..

[43] Whiteley S.L., Weisbecker V., Georges A., Gauthier A.R.G., Whitehead D.L., Holleley C.E. (2018). Developmental asynchrony and antagonism of sex determination pathways in a lizard with temperature-induced sex reversal. Sci. Rep..

[44] Gershon E., Dekel N. (2020). Newly identified regulators of ovarian folliculogenesis and ovulation. Int. J. Mol. Sci..

[45] Chen K., Li S., Xiang J., Sagi A., Li F. (2021). Transcriptome analysis reveals the endocrine regulation on the expression of iag in *Litopenaeus vannamei*. J. Mar. Sci. Eng..

[46] Seufert W., Jentsch S. (1990). Ubiquitin-conjugating enzymes UBC4 and UBC5 mediate selective degradation of short-lived and abnormal proteins. EMBO J..

[47] Wing S.S., Bédard N., Morales C., Hingamp P., Trasler J. (2005). A novel rat homolog of the *Saccharomyces cerevisiae* ubiquitin-conjugating enzymes UBC4 and UBC5 with distinct biochemical features is induced during spermatogenesis. Mol. Cell. Biol..

[48] Yokota N., Harada Y., Sawada H. (2010). Identification of testis-specific ubiquitin-conjugating enzyme in the ascidian *Ciona intestinalis*. Mol. Reprod. Dev..

