# Supplementary material for: The First Identification of Homomorphic XY Sex Chromosomes by Integrating Cytogenetic and Transcriptomic Approaches in Plestiodon elegans (Scincidae)
Source: Genes (Basel). 2024 May 23;15(6):664. doi: 10.3390/genes15060664 (PMC11203037; doi:10.3390/genes15060664)
Supplement: Supplementary file 1 [file genes-15-00664-s001.zip › Supplemental Figures.pdf]

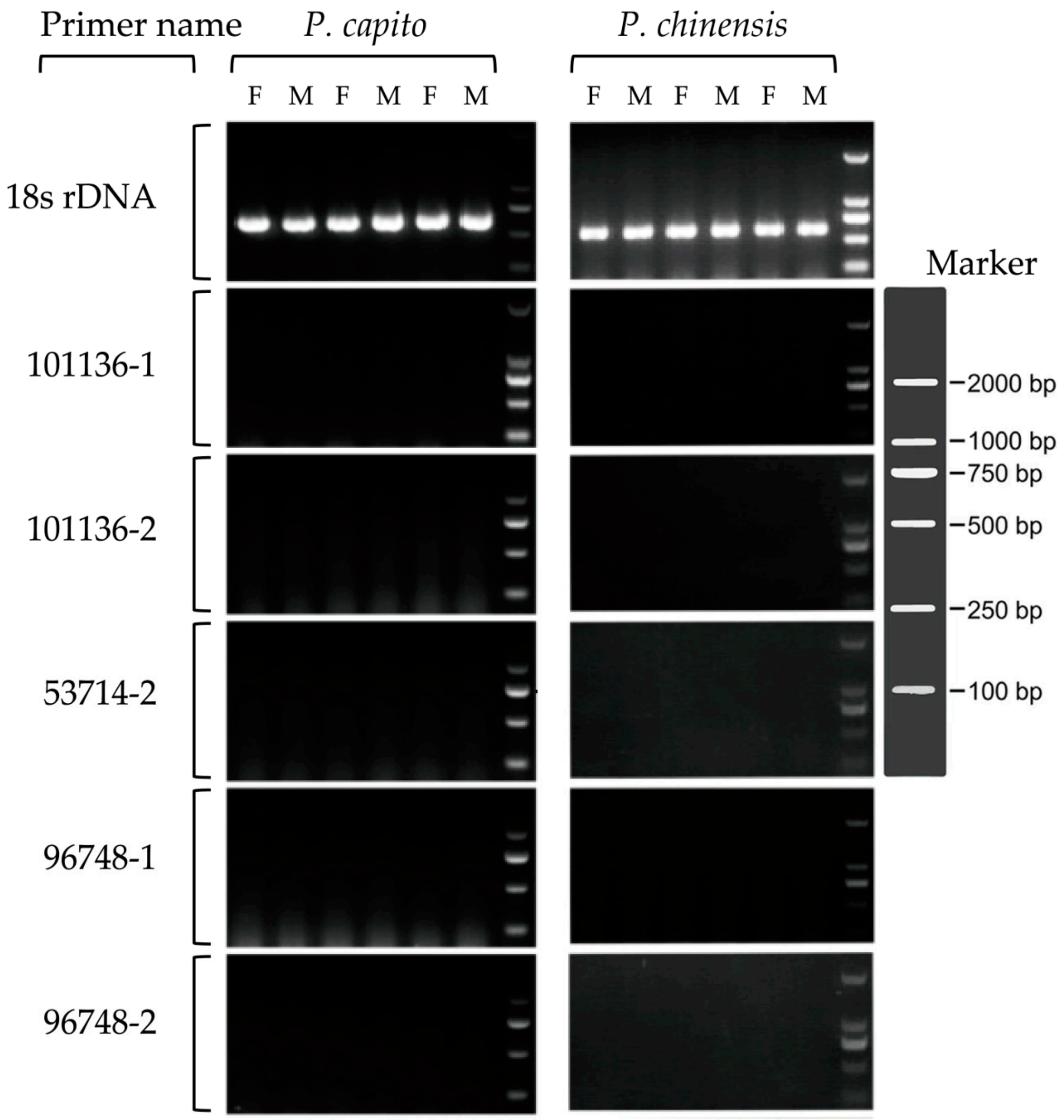

Figure S1: The Y-chromosome markers of *P. elegans* cannot be amplified in P. capito and *P. chinensis*. 18s rDNA as control.

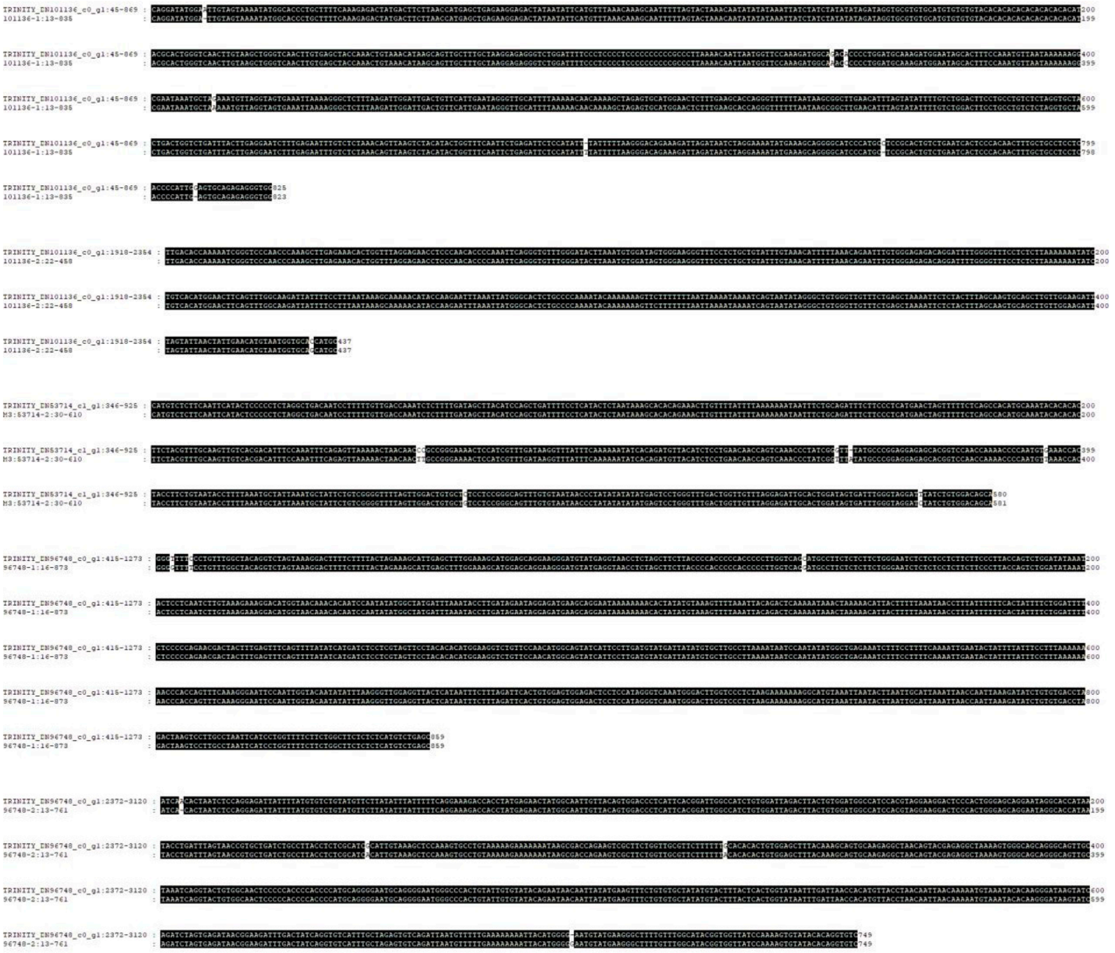

Figure S2: Sequence alignment results of Y-chromosome markers with Y-linked genes, respectively.
